# Iodine Intake and Thyroid Function in Pregnant Women in a Private Clinical Practice in Northwestern Sydney before Mandatory Fortification of Bread with Iodised Salt

**DOI:** 10.1155/2012/798963

**Published:** 2012-11-05

**Authors:** Norman Blumenthal, Karen Byth, Creswell J. Eastman

**Affiliations:** ^1^Department of Obstetrics and Gynaecology, Blacktown Hospital and Norwest Private Hospital, 9 Norbrik Drive, Bella Vista 2153, Sydney, NSW, Australia; ^2^NHMRC Clinical Trials Centre, Faculty of Medicine, 92 Parramatta Road, Camperdown, Sydney, NSW 2050, Australia; ^3^International Council for Control of Iodine Deficiency Disorders, Sydney Medical School, University of Sydney, Sydney, NSW, Australia; ^4^Sydney Thyroid Clinic, Westmead Specialist Centre, Suite 8, 16-18 Mons Road Westmead, NSW 2145, Australia

## Abstract

*Aim*. The primary objective of the study was to assess the iodine nutritional status, and its effect on thyroid function, of pregnant women in a private obstetrical practice in Sydney. *Methods*. It was a cross-sectional study undertaken between November 2007 and March 2009. Blood samples were taken from 367 women at their first antenatal visit between 7 and 11 weeks gestation for measurement of thyroid stimulating hormone (TSH) and free thyroxine (FT4) levels and spot urine samples for urinary iodine excretion were taken at the same time as blood collection. *Results*. The median urinary iodine concentration (UIC) for all women was 81 **μ**g/l (interquartile range 41–169 **μ**g/l). 71.9% of the women exhibited a UIC of <150 **μ**g/l. 26% of the women had a UIC <50 **μ**g/l, and 12% had a UIC <20 **μ**g/l. The only detectable influences on UIC were daily milk intake and pregnancy supplements. There was no statistically significant association between UIC and thyroid function and no evidence for an effect of iodine intake on thyroid function. *Conclusions*. There is a high prevalence of mild to moderate iodine deficiency in women in Western Sydney but no evidence for a significant adverse effect on thyroid function. The 6.5% prevalence of subclinical hypothyroidism is unlikely to be due to iodine deficiency.

## 1. Introduction

Iodine deficiency has been well documented in many parts of the developing world and surprisingly it has reemerged in Australia despite various programs to reduce its incidence [1, 2]. Urinary iodine levels, which are used as a measure of dietary iodine intake, have declined over the past decade, and Australia is now defined by the World Health Organization (WHO) criteria as a mildly iodine-deficient country [3]. The physiological importance of iodine is its central role in maintaining normal thyroid function. Inadequate iodine supply to the thyroid gland will result in decreased thyroid hormone synthesis and enlargement of the gland. During pregnancy iodine intake needs to be increased to meet the demands upon the thyroid gland to boost thyroid hormone production by up to 50% more than preconception requirements, to ensure a supply of thyroxine and iodine to the foetus and to compensate for increased renal iodide clearance [4] 

Iodine deficiency in an asymptomatic young female population may have a negative impact on thyroid function and neonatal outcome, as infants born in areas of moderate to-severe iodine deficiency suffer from a variety of neurodevelopmental disorders caused by irreversible brain damage in utero [5, 6], but adverse effects from mild maternal iodine-deficiency remain less certain. The incidence of neonatal hypothyroidism is also increased in an iodine deficient population, and there is evidence to suggest that severe iodine deficiency may increase rates of miscarriage and stillbirths [6]. 

There is an increasing body of information confirming a high prevalence of mild-to-moderate iodine deficiency in pregnant women in the Australian State of New South Wales (NSW), Victoria, and Tasmania, as evidenced by reduced median urinary iodine concentrations in a number of sporadic studies. [3, 7–11]. None of these studies in pregnant women have examined thyroid function or thyroid volume so it is unknown if iodine deficiency documented in pregnant Australian women has any significant adverse effect on thyroid function. 

The aim of this study was to assess the iodine nutritional status, and its effect on thyroid function, in pregnant women in a private obstetrical practice in metropolitan Sydney. We also wanted to determine if there was an association between urinary iodine concentration, the intake of specific iodine-rich foods, and the use of vitamin and mineral supplements taken by the pregnant women. Analysis of supplements was not undertaken to confirm iodine content reported  on  the  label.

## 2. Materials and Methods 

### 2.1. Participants and Setting

This study was performed in a private practice setting in North Western Sydney between November 2007 and February 2009, where 367 new consecutive antenatal patients were reviewed in their first trimester. At the time of initial booking with the private clinic, between 7 and 11 weeks gestation, routine antenatal investigations were performed. At the same time thyroid function tests, thyroid antibodies, and urinary iodine concentrations were measured. The patients answered questions in relation to their dietary habits, vitamin and mineral supplementation, and family history of thyroid disease. The personal details of the participants are given in Table 1. All women consented to have their blood and urine samples assessed for this study and freely provided the historical information. Women with known thyroid disease were excluded from the study. The study has been approved by the Ethics Committee of the Sydney West Area Health Service.

### 2.2. Urinary Iodine Concentration (UIC), Serum-Free T4, Free T3, and Thyroid-Stimulating Hormone (TSH) Measurements

Iodine concentrations were measured on spot samples of urine by inductively coupled plasma mass spectrometry (ICPMS) in Laverty pathology laboratories in Sydney. Analytical details of the ICPMS method have previously been published (3). Serum TSH concentrations were measured by a chemiluminescent immunoassay on the ADVIA Centaur platform (Bayer Health Care). Serum-free T3 and T4 concentrations were also measured on the ADVIA Centaur platform by a chemiluminescent immunoassay method. For serum TSH the detection limit was 0.01 mIU/L, and intraassay coefficients of variation (CV) were 2.48 and 2.44% at TSH concentrations of 0.74 mIU/L and 5.65 mIU/L, respectively. Between assay % CV varied from 3.2 to 5.9%. The intra-assay and inter-assay CVs for FT4 at 13.9 pmol/L were 2.31% and 3.03%, respectively. Reference intervals provided by the laboratory for serum TSH, FT4, and FT3 in euthyroid adults were from 0.5 to 4.5 mIU/L, 10 to 20 pmol/L, and 3.5 to 6.0 pmol/L, respectively. This laboratory did not provide a specific reference range for pregnancy.

### 2.3. Questionnaire

All patients were asked to state their parity, level of education and ethnicity. A personal history or family history of thyroid dysfunction was obtained, and dietary questions were asked in relation to servings of milk and dairy products per day and portions of fish per week over the preceding weeks of the pregnancy. The use of iodised or noniodised salt was determined and also the use (if any) of vitamin and mineral supplementation, including the brands used.

### 2.4. Statistical Analysis

The statistical software package SPSS Version 17 was used to analyse the data. Two-tailed tests with a significance level of 5% were used throughout. Chi-squared or Fisher's exact tests, as appropriate, were used to test for association between categorical variables. The Mann-Whitney test or Kruskal-Wallis nonparametric analysis of variance were used to test for differences in the distribution of continuous variables by group. Spearman rank correlation (r) was used to quantify the degree of association between continuous and ordered categorical variables.

## 3. Results

### 3.1. Urinary Iodine

**Figure 1:**
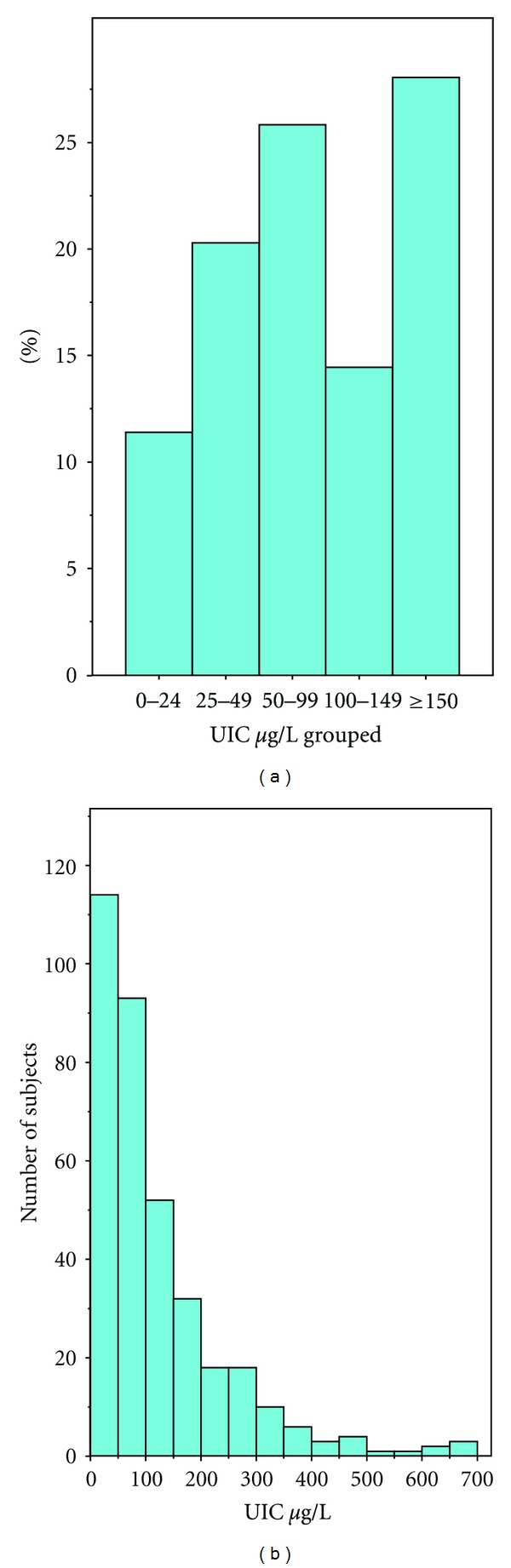
Frequency distribution of UIC in all women.

The median urinary iodine concentration (UIC) for all women (*n* = 367) was 81 *μ*g/L with an interquartile range of 41 to 169 *μ*g/L. A histogram illustrates the distribution of UIC in Figure 1. By conventional WHO UIC standards for a nonpregnant, nonlactating adult population (12), 58% of the women in this study were iodine deficient, comprising 26% with UIC < 50–100 *μ*g/L,, 20% with a UIC 20–49 *μ*g/L, and 12% <20 *μ*g/L. By current WHO standards for pregnant women, 71.9% of the women were iodine deficient having a UIC of less than 150 *μ*g/L (13).  Table 2 sets out the median and interquartile range of UIC by subgroup. There was a statistically significant association between UIC and vitamin supplement (*P* < 0.001). Supplements, during the period of the study, known to contain iodine include Blackmores and Fabfol preparations. In particular, the median UIC of those not taking an iodine supplement was 72 *μ*g/L (IQR 39 to 141), compared with 115 *μ*g/L (from IQR 48 to 213) for those taking a supplement containing iodine (*P* = 0.001). There was no statistically significant association between UIC and parity, ethnicity, and history of thyroid disease. Although there was a significant association between UIC and educational level (*P* = 0.002), there was no significant rank correlation between UIC and increasing educational level (*r* = −0.054, *P* = 0.307). With respect to diet, there was no significant association between UIC, use of iodised salt, dairy or seafood intake. However, there was a significant association between UIC and milk intake (*P* = 0.035). 

### 3.2. Serum TSH and FT4 Concentrations

**Figure 2:**
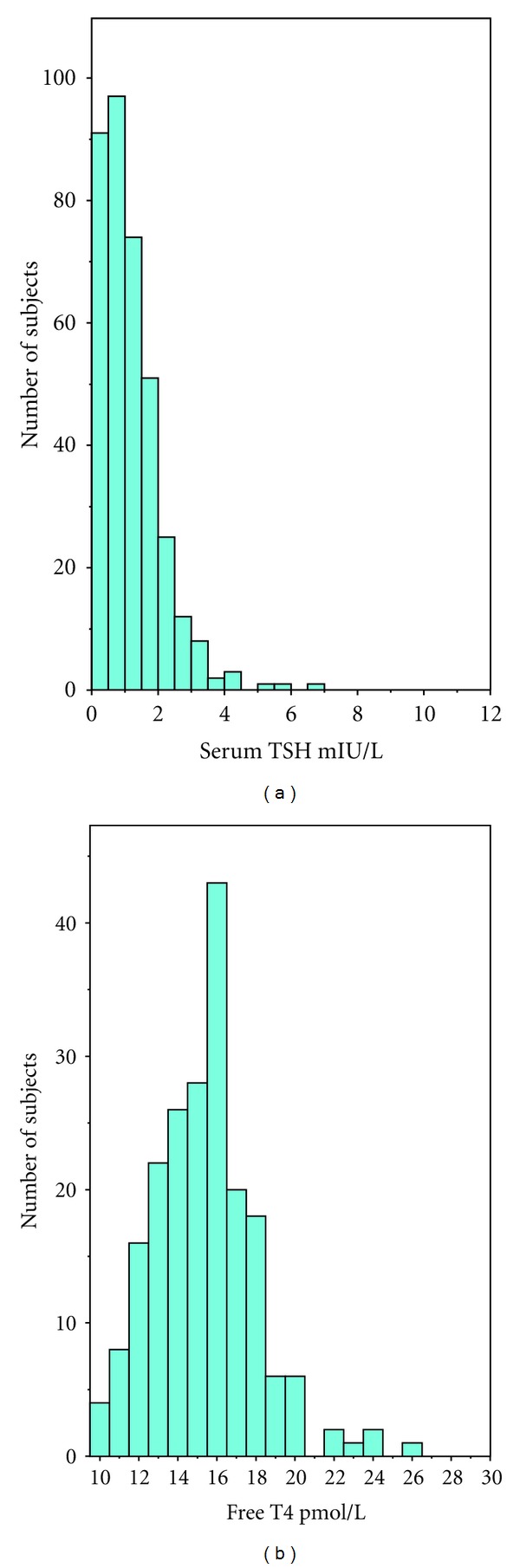
Frequency distributions of serum TSH and Free T4 concentrations.

Table 3 sets out the mean (SD), median, 2.5th, and 97.5th percentiles for the distributions of UIC, TSH and Free T4 concentrations. The distributions for TSH, and Free T4 are illustrated in Figure 2. The mean TSH level was 1.17 mIU/L ±1.11 (SD), and median TSH level was 1.19 mIU/L. 6.5% of the women had a serum TSH >2.5 mIU/L.

There was no significant association between serum TSH and UIC levels (*r* = 0.049, *P* = 0.352), nor between Free T4 and UIC levels (*r* = 0.039, *P* = 0.586). Considering UIC, TSH and Free T4 concentrations as continuous variables, there was no statistically significant association between UIC and either TSH and Free T4 (Spearman rank correlations; 0.049, *P* = 0.352 and 0.039, *P* = 0.586, resp.). When UIC levels are grouped into categories of <50 *μ*g/L, 50–99 *μ*g/L and >100 *μ*g/L, there is no statistically significant evidence of differences in TSH levels or Free T4 levels between these UIC groups (Kruskal Wallis nonparametric ANOVA, *P* = 0.246 and *P* = 0.586, resp.). Similarly, there was no significant evidence of differences in TSH and Free T4 levels in those with UIC  <50 *μ*g/L compared with those with UIC >100 *μ*g/L, *P* = 0.312, and *P* = 0.514, respectively. 

However, there was a significant inverse association between serum TSH and Free T4 levels (*r* = −0.490, *P* < 0.001). 

## 4. Discussion

There was a high overall prevalence of iodine deficiency of 72%, in our population of pregnant women resident in Western Sydney, with 32% suffering from moderate-to-severe deficiency. The median urinary iodine level of 81 *μ*g/L was similar to the level we found in a pregnant population in Sydney approximately 10 years ago indicating that the situation has not improved despite publicity in the medical and lay press about iodine deficiency [7]. Similar reports of iodine deficiency have come from Victoria and Tasmania where the degree of iodine deficiency in pregnant women may be even worse than NSW [9, 11]. Extrapolating from the urinary iodine excretion to calculate daily intake [6], it is evident that these pregnant women are only taking, on average, a little more than half (132 *μ*g) the recommended daily intake (RDI for pregnancy of 250 *μ*g per day [12].

While we are not certain how much reliance one can put on self-reported food and mineral supplement intakes without specific quantitative data, there were some interesting associations between what the participants reported and the measurement of iodine concentrations in their urine. The only food group appearing to influence the urinary iodine level was the intake of milk. It is surprising that other rich sources of iodine such as iodised salt and regular seafood intake did not influence the urinary iodine level. By contrast, there was a highly significant increase in urinary iodine levels in women who were taking a pregnancy supplement. However, the median UIC in the women taking supplements was 115 *μ*g/L, still well below the cutoff level of 150 *μ*g/L, consistent with the RDI for pregnancy of 250 *μ*g per day. It is clear that many of the popular pregnancy supplements either do not contain any iodine, or the amounts are less than what is required to optimise iodine intake for pregnant women. In a recent study Charlton et al. found a median UIC of 87.5 *μ*g/L in a small sample of women attending an antenatal clinic in Wollongong [13]. 59% of the women were taking a pregnancy supplement of which 35% contained iodine. Their findings were similar to ours in that the median UIC in the women taking a pregnancy supplement was significantly increased to 139 *μ*g/L, but still below the recommended level of 150 *μ*g/L. As we have not analysed the iodine content of the supplements taken by our patients we have been unable to verify the quality of these products.

The shortcomings of the study are firstly that it comprised a relatively homogeneous population of women most of whom were well educated and able to afford private obstetric care. In addition the data obtained by questionnaire regarding food intakes and supplements relied upon recall and could not be independently verified. To our knowledge this is the first study of a pregnant population in Australia measuring iodine excretion levels and examining possible negative consequences on thyroid function. As the majority of women were only mildly iodine-deficient significant changes in thyroid function were not anticipated. Measurement of serum TSH is considered the first line test in the laboratory assessment of thyroid function. While the serum TSH level is normally stable in euthyroid adults, it undergoes dynamic change during pregnancy as the thyroid responds to the challenge of having to dramatically increase thyroid hormone production in the first and second trimesters. Unfortunately, TSH reference ranges provided by most laboratories in Australia have not been derived from pregnant populations. In a recent study by Gilbert et al. from Western Australia [14], examining thyroid function during the first trimester of pregnancy in a large sample (excluding women with positive thyroid autoantibodies), they reported a reference range for serum TSH of 0.4 to 4.0 mIU/L and for serum Free T4 of 9.0 to 19 pmol/L. They did not assess iodine nutritional status of their population. Our data, which does not exclude women with positive thyroid autoantibodies, provide similar results with a 2.5 to 97.5 percentile range for serum TSH and Free T4 of 0.03 to 3.4 mIU/L, and 10.0 to 20.5 pmol/L, respectively. Thus, despite the presence of mild iodine deficiency in our pregnant population, it is reassuring that we could not find any evidence for disturbed thyroid function as a consequence of the iodine deficiency. Of course this does not exclude an effect on the thyroid that may be detected by more sensitive measures such as serum thyroglobulin and thyroid volume changes during pregnancy. The fact that 6.5% of the women studied had serum TSH levels greater than 2.5 mIU/L identifies these women as suffering from gestational subclinical hypothyroidism [15]

In summary, mild-to-moderate iodine deficiency is common in pregnant women attending a private obstetrical practice in Western Sydney, but there is no evidence of adverse effects in thyroid function from the level of iodine deficiency in the population studied. Urinary iodine excretion levels have not changed substantially over the past decade despite attempts to improve iodine nutrition in the population at risk [16]. Since this study was completed, it has been mandatory in Australia to replace all salt used in bread making with iodised salt [17]. It is estimated that this strategy would increase mean iodine intake by 46 ug/day and reduce the proportion of nonpregnant women with inadequate intakes from 59% to 9%, but most pregnant women will remain iodine deficient. Iodine supplementation has been recommended as one of the means to achieve improved iodine nutrition in pregnant Australian women [18]. The estimated daily iodine supplement required to provide sufficient iodine intake for pregnant and breastfeeding women has been calculated to be in the range of 100–150 ug/day [19].

## Figures and Tables

**Table 1:** Summary statistics for the 367 patients studied.

Mean age (SD) years	32.0 yrs	(4.3) yrs
Parity	*n*	(%)
Primiparas	145	(40%)
Multiparas	222	(60%)
Highest level education		
School certificate	92	(25%)
Higher school certificate	100	(27%)
Tertiary	175	(48%)
Ethnicity		
Australian born	294	(79.3%)
European	13	(3.5%)
Asian	9	(2.5%)
Filipino	4	(1.1%)
Other	45	(12.3%)
Not available	5	(1.4%)

**Table 2:** Median and quartiles for UIC by subgroup and *P* value for test of homogeneity across subgroups.

Variable	Values taken	(Percent)	Median	Percentile 25	Percentile 75	*P* value
Parity	Primiparas	(40%)	85	41	175	0.736
	Multiparas	(60%)	80	41	161	

Education	School certificate	(25%)	105	52	231	0.002
	Higher school certificate	(27%)	64	33	117	
	Tertiary	(48%)	86	42	166	

Ethnicity	Australian	(79.3%)	78	41	156	0.582
	European	(3.5%)	81	39	178	
	Asian	(2.5%)	80	44	185	
	Filipino	(1.1%)	94	41	177	
	Other	(12.3%)	114	56	180	

History of thyroid disease	Familial and patient	(2.2%)	91	51	183	0.351
	Familial	(14.9%)	90	43	226	
	Patient	(3.3%)	106	72	244	
	Nil	(79.6%)	79	39	156	

Vitamin supplements	Nil	(28.3%)	69	38	136	<0.001
	Elevit	(31.9%)	71	39	120	
	Fefol	(0.3%)	32	32	32	
	Blackmores (iodine 150 *μ*g)	(27.8%)	123	61	226	
	Fabfol (iodine 150 *μ*g)	(4.7%)	60	31	78	
	Other	(6.9%)	123	59	260	

Vitamin supplement containing iodine	No	(67.5%)	72	39	141	0.001
	Yes	(32.5%)	115	48	213	

Iodised salt	No	(78.6%)	81	39	162	0.396
	Yes	(21.4%)	81	47	185	

Milk (grouped)	Nil	(13.0%)	71	39	180	0.035
	<1 serve/day	(23.8%)	77	31	126	
	1 serve/day	(42.3%)	75	45	155	
	>1 serve/day	(21.0%)	105	59	208	

Dairy (grouped)	Nil	(19.1%)	101	47	273	0.078
	<1 serve/day	(22.2%)	76	41	154	
	1 serve/day	(40.7%)	78	34	149	
	>1 serve/day	(18.0%)	87	42	129	

Fish (grouped)	Nil	(29.8%)	72	41	159	0.764
	<1 serve/day	(15.2%)	83	51	120	
	1 serve/day	(39.1%)	89	38	193	
	>1 serve/day	(16.0%)	87	47	179	

Overall		*N* = 367	81	41	169	

**Table 3:** Mean, SD, median, 2.5th, and 97.5th percentiles for TSH, Free T4, and UI concentrations.

	Mean	Standard deviation	Median	Percentile 2.5	Percentile 97.5
TSH (mIU/L)	1.17	(1.11)	0.98	0.03	3.40
T4 (pmol/L)	15.4	(2.7)	15.0	10.0	20.5
Urinary iodine (*μ*g/L)	134.4	(190.0)	81.0	9.2	508.0
